# Are surgeons and anesthesiologists lying to each other or gaming the system? A national random sample survey about “truth-telling practices” in the perioperative setting in the United States

**DOI:** 10.1186/s13037-015-0080-7

**Published:** 2015-11-10

**Authors:** Michael Nurok, Yuo-yu Lee, Yan Ma, Anthony Kirwan, Matthew Wynia, Scott Segal

**Affiliations:** Cardiac Surgery Intensive Care Unit, Division of Cardiothoracic Surgery, Cedars-Sinai Medical Center Heart Institute, 127 San Vicente Blvd, Suite 3100, Los Angeles, CA 90048 USA; Hospital for Special Surgery, New York, USA; George Washington University, Washington, DC USA; Department of Anesthesiology, Brigham and Women’s Hospital, Boston, MA USA; Center for Bioethics and Humanities, University of Colorado Anschutz Medical Campus, Aurora, CO USA; Department of Anesthesiology, Wake Forest School of Medicine, Winston-Salem, NC USA

**Keywords:** Teamwork, Communication, Professionalism, Truth-Telling, Perioperative

## Abstract

**Background:**

The perioperative setting demands strong teamwork to ensure safe patient care, but anecdotally surgeons and anesthesiologists are not always fully truthful with each other. The present study sought to determine the frequency of misrepresentation of the truth in the perioperative setting.

**Methods:**

Direct mailed survey in the United States about misrepresenting information to colleagues in a national random sample of 1130 anesthesiologists and 1130 surgeons.

**Results:**

Reflecting the sensitive nature of these questions, only 252 (11 %) surveys were returned-128/1130 by anesthesiologists and 124/1130 by surgeons. While modest numbers of both anesthesiologists (34/128, 27 %) and surgeons (8/124, 7 %) acknowledged misreporting information at least once per month, misreporting was considerably more common among responding anesthesiologists. Among anesthesiologists the majority (68 %) were concerned that surgeons misreported information to them once a month or more often, though only 8 % of surgeons shared reciprocal concerns. More than a third of responding anesthesiologists (36 %) reported having seen their teachers misreport information to surgeons during their training.

**Conclusions:**

These findings, though preliminary due to the small sample, raise concerns about a possible culture of misrepresentation, passed on between generations, in some perioperative environments. Misreporting of information should be examined in more detail and addressed at local levels whenever it is found. Further research is required to determine if the reported behaviors represent routine gaming of perioperative care systems or deliberate and intentional deception. Strategies aimed at fostering conditions in which open honest communication can thrive should be investigated.

## Background

There is increasing attention to safety culture, teamwork and communication practices in medicine [1, 2]. This focus has lead to large initiatives focused on effective communication skills [3]. As a result of such initiatives, the contribution of dysfunctional behavior in the perioperative setting is increasingly being scrutinized as a potential source of adverse outcomes and healthcare worker job dissatisfaction [4, 5]. Among these dysfunctional behaviors may be intentional physician-to-physician deception.

The phenomenon of intentional misrepresentation of the truth among physicians is little studied. Physicians occasionally misrepresent the truth to insurance companies to gain coverage for their patients [6–8], and there is a modest literature on the evolution of physician attitudes towards misrepresentation to patients with poor prognoses [9]. But almost nothing is known about whether physicians ever intentionally deceive each other in the course of patient care.

Anecdotally, anesthesiologists and surgeons may misrepresent the truth to each other in the course of perioperative care, such as by misreporting blood losses, the urgency of a case, or the volume of fluids given. Research has shown that surgeons are often inaccurate in their prediction of the duration of cases [10–12], but it is unknown whether this inaccuracy is intentional or not. Similarly, documentation in the anesthetic record can vary considerably when record keeping is automated as compared to manual documentation by an anesthesia provider [13, 14], but the reasons for these discrepancies and whether they are intentional or not are also unknown.

## Methods

To test the hypothesis that some degree of intentional misrepresentation might occur among physicians in the perioperative setting we conducted a pilot survey of anesthesiologists and surgeons nationally.

After obtaining approval from the Partners Human Research Committee^1^ we mailed a self-administered survey to 1130 anesthesiologists and 1130 surgeons drawn from the American Medical Association (AMA) Masterfile, which includes all physicians practicing in the United States. After removing ineligible recipients, the sample of anesthesiologists was 1084 and for surgeons it was 1090. As an incentive, respondents were entered into a raffle to win an iPod. Six months after the initial mailing a follow-up post-card was mailed requesting that the original survey be returned or directing respondents to a secure website to enter responses.

To assess the frequency of misreporting information among anesthesiologists and surgeons, each survey included a set of potential facts (such as the amount of blood loss, the urgency of the case, or minor complications not apparent to the other physician) that could be misreported.  Each list was tailored to anesthesia or surgical practice (complete lists are available from the authors).  Survey recipients were asked to report how often, if ever, they had “knowingly mis-reported (or chosen not to report) the following intra-operative information to any member of the surgical/anesthesia team?” Responses on a 6-point likert scale ranged from 1) daily, 2) once or twice a week, 3) once or twice a month, 4) once or twice a year, 5) less than once a year, 6) and never.

Next, a list of potential justifications for misreporting was presented and respondents were asked “How important are the following possible justifications for deciding to mis-report (or not report) information to the surgical/anesthesia team.” Responses on a 5-point likert scale ranged from 1) very unimportant, 2) somewhat unimportant, 3) somewhat important, 4) very important, and 5) not applicable or I never mis-report. Respondents were also asked “in the last year, how often have you been concerned that your surgical/anesthesia colleagues have misrepresented the truth to you?” with 6 response options ranging from”daily” to “never”.

Respondents were informed in the cover letter that their responses would remain strictly confidential and that the Partners Human Research Committee approved the study protocol. Because of the sensitive nature of the survey questions, each subject was randomly assigned a unique identifier. Mailings were prepared in Chicago but return-mailed to the Boston-based investigators. The key linking unique identifiers to subjects was kept in Chicago, thereby preventing the Boston based team from linking responses to subjects.

Survey results were tabulated and demographics of responders and non-responders were compared by cross-indexing the unique identifier to demographic data in the AMA Physician Masterfile.  Descriptive statistics were presented for demographics and for survey results. Continuous variables were summarized in means and 95% confidence intervals while categorical variables were reported as frequencies and proportions. Demographics and survey responses of responders vs. non-responders, and anesthesiologists vs. surgeons were compared using two-sample Student’s t-test or chi-square test. All tests were two sided with a significance level of 0.05.

## Results and discussion

Given the highly sensitive nature of the questions we asked, it is not surprising that this pilot survey generated a relatively low response rate (11 %, 252/2260); 128/1084 by anesthesiologists and 124/1090 by surgeons. By the same token, we suspect our exploratory findings on the frequency of anesthesiologist-surgeon deception may be conservative, given the socially undesirable nature of admitting to intentional deception of colleagues. More importantly, despite being far from definitive, our results raise provocative questions about truth-telling practices among physicians in the perioperative setting.

There were no significant differences between respondents and non-respondents for age or years since graduation, although more male surgeons did not respond to the survey (*P* = 0.009) (Table 1). Amongst respondents, there were no significant differences between anesthesiologists and surgeons for age, sex, years in practice, hospital size, English as a first language, or self reported religious status (Table 2).

**Table 1:** Respondents vs non-respondents demographics from AMA Masterfile

		Anesthesiologist	Surgeon
Age, mean (95 % CI)	Respondents	51.6 (49.8–53.3)	53.4 (52.8–54.2)
	Non-respondents	51.6 (50.9–52.2)	53.5 (51.1–55.8)
	*p* value	*P* = 0.971	*P* = 0.958
Male sex	Respondents	99/127 (77.95 %)	69/85 (81.2 %)
	Non-respondents	799/1002 (79.7 %)	942/1044 (90.2 %)
	*p* value	*P* = 0.638	*P* = 0.009
Years since graduation, mean (95 % CI)	Respondents	24.0 (22.2–25.9)	26.9 (24.4–29.4)
	Non-respondents	24.5 (23.9–25.2)	26.95 (26.2–27.7)
	*p* value	*P* = 0.624	*P* = 0.960

**Table 2:** Respondent demographics–self reported

	Anesthesiologist	Surgeon	
	* N* = 128	*N* = 124	*p* value
Age, mean (95 % CI)	50.95 (49.47–52.43)	52.51 (50.62–54.39)	0.2003
Male sex	98 (77 %)	103 (83 %)	0.424
Years of practice, mean (95 % CI)	19.12 (17.62–20.62)	20.76 (18.80–22.72)	0.1881
English first language	110 (86 %)	116 (94 %)	0.059
Hospital type			
Academic <500 beds	18 (14 %)	16 (14 %)	0.486
Academic >=500 beds	25 (20 %)	15 (13 %)	
Non-academic <500 beds	71 (56 %)	75 (63 %)	
Non-academic > =500 beds	14 (11 %)	13 (11 %)	
Religious			
Very	11 (9 %)	21 (13 %)	0.084
Moderately	40 (31 %)	42 (33 %)	
Slightly	44 (34 %)	28 (29 %)	
Not	33 (26 %)	31 (26 %)	

Among the 252 respondents to our survey, up to 27 % of anesthesiologists and 7 % of surgeons admitted to misrepresenting the truth to each other at least once a month regarding a number of factors that can influence perioperative care (Tables 3 and 4). The justifications reported typically involved concerns around the adverse consequences of being truthful; in particular, paternalistic concerns about disclosing truthful information to a colleague who might not understand or who would then demand unreasonable steps (Table 5). Such concerns were the drivers of intentional misrepresentation much more often than worries about personal blame, chastisement or legal consequences.

**Table 3:** Most commonly misreported events by Anesthesiologists (a)

Anesthesia actions that impacted vital signs (e.g. a recruitment maneuver)	34 (27 %)
Vital signs different than those measured	25 (20 %)
Volume of Fluid Given	23 (18 %)
Anesthesia related adverse events, regardless of how minor, routine, or seemingly unimportant (eg Chipped tooth or bloddy lip following intubation)	19 (15 %)
Vasopressor	18 (14 %)
Drugs	11 (9 %)
Medication dose	11 (9 %)

^a^Percent respondents ackowledging intentional misreporting of information monthly or more often

**Table 4:** Most commonly misreported events by Surgeons (a)

The estimated length of the surgical portion of a case	8 (7 %)
Intra-operative adverse events, regardless of how minor, or seemingly unimportant	5 (4 %)
Estimated surgical risk	4 (3 %)
The urgency of a case	4 (3 %)
Surgical actions that impacted vital signs (e.g. pushing on the heart, releasing a vascular clamp)	3 (2 %)
A patient’s co-morbidities	3 (2 %)
The estimated length of the surgical portion of a case	3 (2 %)
Your unavailability to do a case	3 (2 %)

**Table 5:** Justifications considered “Very Important” when misrepresentation was acknowledged (*)

Rationale	Anesthesiologist		Surgeon		*p* value
Patient’s Best Interest	11/60	18 %	2/19	11 %	0.685
Information Not Clinically Relevant	49/79	62 %	16/39	41 %	0.031
Already Attending to Issue	40/82	49 %	7/33	21 %	0.007
Counterpart Would Not Understand	27/81	33 %	5/26	19 %	0.172
Counterpart Would Demand Unreasonable Tx	25/82	31 %	8/29	28 %	0.769
Would be Blamed or Chastised	5/78	6 %	0/26	0 %	0.459
Not A Good Time to Discuss	31/82	38 %	8/31	26 %	0.232
Concerned About Legal Consequences	15/71	21 %	10/32	31 %	0.269

*Percent of respondents acknowledging a survey item as a justification for mis-reporting who considered the justification to be “very important”

*N’s are different because more than one item could be acknowledged as a justification

When clinical decision making is based on erroneous information, a cascading series of events can occur, leading to harm to patients and adverse consequences for physicians. This is indicated by the fact that 7 % of responding anesthesiologists and 2 % of surgeons said that a patient had been harmed by their misrepresentation of the truth to a colleague (data not shown in tables). Alternatively adverse consequences may occur to the physician who is misrepresenting or receives misrepresented information. In this survey 1 % of anesthesiologists and 4 % of surgeons reported suffering adverse consequences themselves due to an episode of misrepresentation (data not shown in tables). The nature of harms to patients and consequences to physicians cannot be identified from the present survey and requires further investigation.

The greatest impact of dishonest communication between colleagues in the perioperative setting may be that it damages teamwork among professionals engaged in high-stakes activities.  Candid and open communication and shared decision-making are foundational to optimizing teamwork. Studies have shown correlations between such team skills and actual or potential improved outcomes for patients [15, 16].

What can be learned from these pilot data? First, the motivations for intentional misrepresentation of the truth to colleagues likely represents a spectrum, ranging from gaming of the perioperative care system to optimize the ability to accomplish what physicians feel to be in the best interests of their patient to willful misrepresentation and deception (or lying) with a view to obfuscating facts to obtain personal benefits. Our results suggest the former motivation predominates, though future research should aim to better address the issue of motivation.

Second, the apparent asymmetry of our survey results requires further investigation. Anesthesiologists were both more likely to report misrepresenting facts to surgeons and more likely to be concerned about surgeons misrepresenting facts to them (Table 6). Perhaps of greater concern, more anesthesiologists (36 %) than surgeons (8 %) reported that they had seen their teachers engage in misrepresentation of the truth during their training. It is possible that anesthesiologists are more willing to be honest about misrepresenting issues to surgeons on a survey, or it could be that intentional misrepresentation really is more common within the anesthesia community. This question deserves focused attention, given its potential import for the profession, especially if in some cases deliberate misrepresentation is being taught, explicitly or implicitly, to trainees, perhaps as an anticipatory strategy for controlling or gaming the perioperative environment.

**Table 6:** How often have you been concerned your counterpart is misrepresenting information (in the past year)?

	Anesthesiologist	Surgeon	*p* value
			<0.001
Never	11 (9 %)	72 (59 %)	
Less than once a year	2 (2 %)	16 (13 %)	
Once or twice a year	29 (23 %)	25 (21 %)	
Once or twice a month	51 (41 %)	7 (6 %)	
Once or twice a week	22 (18 %)	2 (2 %)	
Daily	11 (9 %)	0	

Third, we hope these preliminary results will spark more formal study of these issues. This poll was intended to generate conversation and hypotheses. After all, if there is a possibility that misrepresentation is becoming normative, on the assumption that one’s anesthesiology or surgeon counterpart is also engaging in a similar practice, this deserves direct and focused attention. In this regard, our results are especially troubling if our estimates for prevalence are low. In addition, while some might argue that minor misrepresentations comprise a normal social practice, our results also suggest that a potential for harm to patients and physicians exists and that further discussion, exploration and investigation is warranted.

Fourth, with regard to future research, this pilot work suggests that any future survey research on this topic will need to include robust efforts to obtain high response rates; and it is possible that the very sensitive nature of this issue will always preclude obtaining high levels of response. Other approaches to this area of enquiry might yield valuable insights, such as using qualitative data or the examination of automated data collected during surgery.

Finally, while our study focused on misrepresentations between surgeons and anesthesiologists, it is reasonable to be concerned that misrepresentation of medical practice might occur between other health professionals. For example, there are case reports of nursing staff intentionally hastening a patient’s death without explicit authority to do so and in the absence of formal documentation [17]. Residents have also reportedly engaged in sham resuscitations when they believed orders for resuscitation were not warranted [18]. These examples suggest that misrepresentation is probably more widespread in medicine than just between physician colleagues in the perioperative setting.

## Footnotes

1 The Partners Human Research Committee is the Institutional Review Board for Brigham and Women’s Hospital and Massachusetts General Hospital comprising amongst others, ethicists, physicians, and lay persons.
